# Assessment of Bioactive Phenolic Compounds and Antioxidant Activity of Blackberry Wines

**DOI:** 10.3390/foods9111623

**Published:** 2020-11-07

**Authors:** Daniela Amidžić Klarić, Ilija Klarić, Ana Mornar, Natalija Velić, Darko Velić

**Affiliations:** 1 Department of Pharmaceutical Analysis, Faculty of Pharmacy and Biochemistry, University of Zagreb, Ante Kovačića 1, 10000 Zagreb, Croatia; amornar@pharma.hr; 2 Department of Health Ecology, Public Health Brčko DC, R. Dž. Čauševića 1, 76000 Brčko DC, Bosnia and Herzegovina; klaric67@gmail.com; 3 Department of Process Engineering, Faculty of Food Technology Osijek, J. J. Strossmayer University of Osijek, Franje Kuhača 18, 31000 Osijek, Croatia; natalija.velic@ptfos.hr (N.V.); darko.velic@ptfos.hr (D.V.)

**Keywords:** blackberry wine, phenolic compounds, antioxidant activity, blackberries cultivation methods

## Abstract

Blackberry wine is a natural source of bioactive phenolic compounds that have profound antioxidant potential. The objectives of the present research were to assess the phenolic compounds and antioxidant activity of blackberry wines (BW), and to use the chemometric analysis to differentiate among the two groups of samples, i.e., conventional and organic. Fifteen BW samples were analyzed for their total polyphenol index, total polyphenols, total flavonoids, total tannins, total monomeric anthocyanins and antioxidant activity by the appropriate spectrophotometric methods. The concentrations of individual phenolic acids (gallic acid, chlorogenic acid, caffeic acid, *p*-coumaric acid and cinnamic acid) and *trans*-resveratrol were determined by high-performance liquid chromatography. A comparison between the two groups of investigated BW samples revealed a statistically significant difference in the concentration of caffeic acid and *p*-coumaric acid, both being higher in the organic BW samples. Furthermore, the results showed a series of statistically highly significant relationships between the analyzed constituents (caffeic acid and *p*-coumaric acid). The antioxidant activity of the investigated wines was proportional to the concentrations of bioactive phytochemicals.

## 1. Introduction

As secondary plant metabolites, polyphenolic compounds are widespread in fruits and fruit-based products, thus enhancing their nutraceutical potential and functional properties. The development of new functional products containing significant amounts of bioactive compounds (e.g., polyphenolics) that, besides having nutritional value, exhibit positive health effects, has been a steady trend in the food industry for some time [1]. Keen interest in the development of such products has led to the development of appropriate qualitative and quantitative methods of analysis of bioactive compounds, as well as methods of their distinct separation within a complex food matrix, such as fruit wine.

Blackberry wine, a traditional alcoholic beverage of Croatia, is a natural source of minerals and many bioactive phytochemicals, such as polyphenolics, that have a profound antioxidant potential and can play an essential role in the prevention of disease and the promotion of health [2,3,4,5]. During the last decade, much research effort has been devoted to determining the influence of the moderate consumption of fruit wine on overall health [4,6,7,8,9,10]. This research interest has mostly been focused on the protective effect of bioactive compounds against the development of common diseases, such as cardiovascular, cancer, and neurodegenerative [11,12,13]. It has been generally accepted that different oxidation processes play a significant role during the initial steps of the development of the aforementioned diseases. Indeed, reactive oxygen species (ROS), naturally formed during healthy physiological metabolism, can damage structural biological molecules, such as proteins, lipids or deoxyribonucleic acid (DNA). Human metabolism mainly relies on endogenous antioxidants as a defense system. However, this defense system is not sufficient under certain conditions (e.g., stress), and adverse effects still occur. Therefore, increasing dietary intake of exogen antioxidants is recommended in order to improve defense responses against ROS.

As mentioned, blackberry wine is typically rich in plant antioxidants, including polyphenolics, and is expected to exhibit strong antioxidant activity. However, the content and specific phenolic profile of blackberry wine may be influenced by several factors, i.e., mainly, the type and variety of fruit, conditions and techniques of fruit cultivation (e.g., organic or conventional), technological wine production process, wine maturation and storage conditions (temperature, wine pH and sulfite and ethanol content) [1,14,15,16]. The blackberry cultivars most commonly cultivated in Croatia and used for blackberry wine production are Thornless Logan, Black Satin, Tayberry and Thornfree. Furthermore, a large share of cultivated farming in Croatia is based on organic principles, and the resulting fruit wines are denoted as “made of organic blackberry” [1]. Even though there are inconsistencies in research results that deal with the comparison of organic and conventional food products based on their bioactive compound and nutrient contents, when it comes to wines and fruit wines, some authors have reported higher antioxidant activity [17], lower levels of pesticides and ochratoxin A [18], as well as slightly higher levels of quercetin [19], in organic wines.

Until a decade ago, there was a general lack of research on fruit wines. The same applies to data on the phenolics content and antioxidant activity of blackberry wines. In our previous research [3] conducted on commercially available Croatian blackberry wine samples originating from three different geographical regions, we evaluated the content of different polyphenols and antioxidant activity of samples with respect to their geographical region. Furthermore, we evaluated the quercetin content, color and selected physicochemical quality parameters, mineral content, alcohols, volatile compounds and contents of food additives of Croatian blackberry wines produced from organically and conventionally grown blackberries [19,20,21]. This work is a continuation of that research.

The objective of this study was to re-evaluate, from a qualitative and quantitative point of view, the phenolic composition and content, as well as antioxidant activity, of commercially available Croatian blackberry wines. Another objective was to use the obtained results to explore the possibility to differentiate between the investigated samples based on the applied cultivation method of the raw material (conventionally grown or organically grown blackberries) using chemometric analysis.

## 2. Materials and Methods

### 2.1. Chemicals and Standards

All reagents used in this work were of analytical reagent grade or better. Standards of *p*-coumaric acid, chlorogenic acid, and trans-resveratrol were purchased from Sigma–Aldrich (Steinheim, Germany), while caffeic and cinnamic acid were obtained from Fluka BioChemika (Buchs, Switzerland).

Folin–Ciocalteu’s phenol reagent, ferric chloride anhydrous, potassium dihydrogen phosphate, potassium chloride, sodium acetate anhydrous and methylcellulose *Tylose* MH 300 were supplied from Fluka (Buchs, Switzerland), while ammonium sulphate, sodium carbonate anhydrous, phosphoric acid (85% (*w*/*w*), density = 1.70 g/cm^3^, p.a.) and formaldehyde solution 35% (p.a.) were from Kemika (Zagreb, Croatia). Potassium persulfate, dipotassium phosphate and hydrochloric acid fuming (37% (*w*/*w*), density 1.19 g/cm^3^, p.a.) were provided by Merck (Darmstat, Germany).

Trichloroacetic acid and standard of gallic acid were acquired from Riedel–de Haën (Seelze, Germany), while potassium ferricyanide and BHA (butylated hydroxyanisole) were from HiMedia (Mumbai, India). DPPH• (2,2-diphenyl-1-picrylhydrazyl•, 95%) and ABTS^•+^ (2,2′-azino-bis- [3-ethylbenthiazoline-6-sulfonic acid]) in the crystallized diammonium salt form were delivered from Sigma–Aldrich (Steinheim, Germany). Acetonitrile, glacial acetic acid, and methanol were purchased from Carlo Erba Reagent (Milano, Italy). Double deionized water (DDW) was used in all experiments (resistivity of 18.2 MΩ cm/25 °C).

### 2.2. Blackberry Wine Samples

The phenolics content and antioxidant activity were evaluated in fifteen blackberry wine samples. The samples were collected directly from the small-scale producers to ensure that all wines were from the same vintage year and stored properly before the collection. All wine samples originated from the continental part of Croatia. Triplicate bottles from the same production batch of each blackberry wine sample were collected, stored at +4 °C, and opened just before the analyses. Based on the cultivation method, the samples were divided into conventional and organic groups. Seven samples comprised the conventional group (CBW 1–CBW 7), while eight (OBW 8–OBW 15) comprised the organic group (wines were produced using blackberries cultivated in line with the EU organic cultivation legislation [22,23]). The alcoholic content, determined according to the OIV standard method, ranged from 9.37 to 14.78% vol [20].

### 2.3. HPLC Determination of Phenolic Acids and Trans-Resveratrol in Blackberry Wine.

The concentration of five phenolic acids (gallic acid, chlorogenic acid, caffeic acid, *p*-coumaric acid and cinnamic acid) and *trans*-resveratrol were determined by HPLC. Chromatographic analyses were conducted on a Dionex chromatographic system (Sunnyvale, CA, USA). The system consisted of an ASI 100 automatic sample injector, a TCC-100 columns oven, a P680 pumping system, a UVD170S Detector and Chromeleon 6.8 software (Dionex, Sunnyvale, CA, USA). The samples were filtered through Minisart RC4, 0.45 µm filters (Sartorius, Göttingen, Germany), which did not retain any of the analytes. After injecting 20 μL of sample, polyphenolic compounds were separated on a Lichrospher 100-RP18 column (250 × 4 mm, 5 µm; Agilent Technologies, Santa Clara, CA, USA) with suitable guard column (LiChrospher^®^ 100, RP18 (5 µm), 4 × 4 mm; Agilent Technologies, Santa Clara, CA, USA).

**Figure 1:**
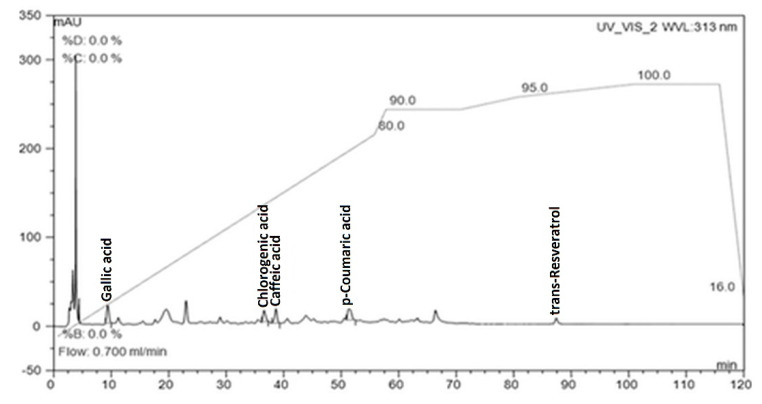
Chromatogram of blackberry wine sample (CBW4) recorded at 313 nm for phenolic acids and *trans*-resveratrol.

The chromatographic conditions were modified based on Gambelli and Santaroni [24]. The mobile phase in gradient elution consisted of (A) acetic acid (2%) and (B) water:acetonitrile:acetic acid (78:20:2). The concentration of eluent B was increased from 0% to 80% over 55 min, and to 90% B at 57 min, 90% B at 70 min, 95% B at 80 min, 100% B at 100 min, 100% B at 115 min, and 0% B at 120 min. Elution was carried out at a flow rate of 0.7 mL/min, and the column was thermostatically controlled to maintain a temperature of 22 °C. During each run, the chromatogram (Figure 1) was recorded at 280 nm (gallic acid, cinnamic acid, and trans-resveratrol), 313 nm (*p*-coumaric acid) and 323 nm (caffeic and chlorogenic acid).

The proposed methods for the determination of phenolic acids and *trans*-resveratrol in samples were validated based on the recommendations of the International Conference on Harmonization (ICH) guidelines [25]. Validations of the methods were carried out for linearity, sensitivity, repeatability and intermediate precision (Table 1).

### 2.4. Spectrophotometric Methods

Spectroscopic analyses were carried out using Lambda 25 UV–Visible spectrophotometer (double–beam) (Perkin–Elmer, Waltham, MA, USA). Data were recorded by Perkin–Elmer’s Spectroscopy Software UV WINLAB (version 2.85.04). Quartz cells with 1.0 cm path length (Perkin–Elmer, Waltham, MA, USA) were used for all samples.

#### 2.4.1. Determination of Total Polyphenol Index (TPI)

TPI was determined spectrophotometrically as described by Organisation Internationale de la Vigne et du Vin (OIV) [26]. The samples were diluted 100 times with DDW, and absorbance was measured at 280 nm. The absorbance spectra were recorded in the working range 190–800 nm with a step resolution of 1 nm. DDW was used for the reference scan.

#### 2.4.2. Determination of Total Polyphenols—Folin–Ciocalteu Index (TPH)

TPH was estimated by Folin–Ciocalteu colorimetric assay based on the procedure described by OIV [26]. The incubation period lasted for 2 h at room temperature, after which the absorbance was measured at 760 nm. Gallic acid was used as a standard. The results were determined from a gallic acid standard calibration curve and expressed as gallic acid equivalents (mg_GAE_/L).

#### 2.4.3. Determination of Total Flavonoids

Flavonoids content was determined by the previously proposed method [27] using formaldehyde for precipitation. The content of flavonoids (mg_GAE_/L), was calculated as the differences between total polyphenols and nonflavonoids.

Briefly, 10.0 mL of the previously filtered sample through a 0.45 μm filter was precipitated into a volumetric flask class A, added 10.0 mL HCl (1 + 3) and 5.0 mL formaldehyde (8 mg/mL). The volumetric flask was well closed, mixed and left for 24 h at room temperature. After that, the reaction mixture was filtered through a 0.45 μm pore diameter filter, and the concentration of residual nonflavonoid polyphenols was determined in the resulting filtrate using the Folin-Ciocalteu reagent [26]. The results were expressed as gallic acid equivalents (mg_GAE_/L).

#### 2.4.4. Determination of Total Tannins

The method is based on the precipitation of tannins with methylcellulose and ammonium sulfate. The tannins content, expressed in mg_GAE_/L, was calculated as the difference between total phenols and nontannins [28].

Briefly, 2.0 mL of the wine sample was pipetted in volumetric flask class A, 1 mL of 0.4% (*w*/*v*) methylcellulose solution and 2 mL of saturated ammonium sulfate were added; then, the volume was brought to 10 mL with DDW. The reaction mixture was shaken well and centrifuged for 10 min at 4000 rpm. Residual nontannin polyphenols in the liquid upper layer were determined using the Folin-Ciocalteu reagent.

#### 2.4.5. Determination of Total Monomeric Anthocyanins (ACY)

ACY was determined using the pH differential method [29]. Blackberry wines were diluted using two different buffers (potassium chloride buffer pH 1.0 and sodium acetate buffer pH 4.5). Absorbance was measured simultaneously at 510 nm and 700 nm, and ACY were expressed as mg malvidin-3-glucoside per L of blackberry wine. A molar extinction coefficient of 28,000 L/(cm mol) was used for malvidin-3-glucoside (molecular weight of 463.3 g/mol).

#### 2.4.6. DPPH Method

The capacity of blackberry wines to scavenge the ‘stable’ free radical 2,2-diphenyl- 1-picrylhydrazyl• (DPPH•) was monitored according to the adjusted method of Hatano et al. [30], with some slight modifications. The samples were diluted with methanol and an aliquot of 4 mL of various concentrations of sample added to a methanolic solution of DPPH• (1 mM, 0.5 mL). The mixture was mixed (using vortex) for 15 s and then left in the dark at room temperature for 30 min. The absorbance of the resulting solution at 517 nm was used to determine the concentration of remaining DPPH•. A methanolic solution of 2 mg of BHA dissolved in 4 mL of methanol and 0.5 mL of the DPPH• solution was chosen as a background correction.

#### 2.4.7. ABTS Method

Free radical scavenging activity of samples was also determined by ABTS^•+^ radical cation decolourization assay [31]. The ABTS^•+^ monocation radical solution was prepared by mixing equal volumes of a 7 mM ABTS^•+^ stock solution and a 2.45 mM potassium persulfate solution, both in DDW. The mixture was stored in the dark at room temperature for 12 h before use. After that, the resulting solution was diluted with DDW to get an absorbance of 0.700 ± 0.025 at 730 nm. Briefly, 0.5 mL of ABTS^•+^ solution was added to aliquot of 3 mL of blackberry wine diluted in DDW at different concentrations. The reaction mixture was incubated at room temperature for 30 min and the absorbance was immediately recorded at 730 nm. The percentage inhibition was calculated for each concentration relative to a blank absorbance (DDW).

For DPPH and ABTS methods, the IC_50%_ values were calculated as the concentration of blackberry wine required for 50% inhibition [3].

#### 2.4.8. Reducing Power Assay (RPA)

RPA was determined according to the method of Oyaizu [32] and Yen & Chen [33], with some slight modifications. Briefly, 0.1–1.0 mL of each investigated blackberry wine was diluted up to 25 mL with DDW in glassware (grade A). 2.5 mL of a 0.2 M phosphate buffer (pH 6.6) and 2.5 mL of a 1% (*w*/*v*) solution of potassium ferricyanide were added in 1.0 mL of prepared aliquot taken in a 10–mL test tube. The resulting solution was incubated in a shaking water bath at 50 °C for 20 min. Following this, 2.5 mL of a 10% (*w*/*v*) trichloroacetic acid solution was added, and the mixture was then centrifuged at 1750× *g* for 10 min. A 2.5 mL aliquot of the supernatant was combined with 2.5 mL of DDW and 0.5 mL of a 0.1% (*w*/*v*) solution of ferric chloride. The absorbance of the reaction mixture was read at 700 nm. Increased absorbance of the reaction mixture indicated higher reducing power.

#### 2.4.9. Molybdenum (MoT) Test

The method is based on the reduction of Mo(VI) to Mo(V) by sample and the subsequent formation of green-colored phosphomolybdene(V) complex at acid pH [34].

Briefly, 0.1 mL diluted sample of blackberry wine was added to the test tube, and 3.0 mL of reagent solution (containing 0.6 M sulfuric acid, 28 mM sodium phosphate and 4 mM ammonium molybdate) was added. The test tube was incubated for 90 min at 95 °C. After cooling the test tube at room temperature, the absorbance was measured at 695 nm. The antioxidant activity was expressed as ascorbic acid equivalents (mg_AA_/L) determined from a standard calibration line.

### 2.5. Statistical Methods

All determinations were conducted in triplicate, and the data presented as the means ± standard deviations. All data were firstly tested for normal distribution using the Kolmogorov–Smirnov test. The normally distributed variables were described by the arithmetic mean and standard deviation, while variables not showing a normal distribution were presented by the median and interquartile range.

The univariate characterization of samples based on the blackberries’ cultivation method was carried out using a *t*-test for normally distributed variables and nonparametric statistics (the Mann–Whitney U test) for others. The Pearson product-moment correlation coefficient and Spearman rank correlation coefficient were determined to examine potential relationships between the concentrations of compounds and antioxidant activity.

Principal component analysis (PCA) was used to provide a new set of variables (the lowest possible number of variables) that were calculated in a way that would keep most of the information presented within the original dataset (including quercetin and anthocyanidins (cyanidin and pelargonidin) data from our previous study [19]). Principal components (6 PCs) derived from the original dataset with the cumulative percentage of total explained variance >88.8% were used for further analysis with general discriminant analysis (GDA).

GDA was done using the original dataset and compared with the same analysis done with PCs for the model of polyphenol content and antioxidant activity using a backwards stepwise approach to produce the discriminant model with the least number of variables but still enough statistical power to discriminate significantly between groups and with the GDA with PCs using the all effects model. Also, a GDA model was applied using two variables from the original dataset that were significantly different between the groups. A classification model for group separation was provided for all GDA models. *p* < 0.05 was considered statistically significant and *p* < 0.01 very significant. The statistical package STATISTICA v. 12 from StatSoft^®^ (Tulsa, OK, USA) was used for data analyses.

## 3. Results and Discussion

Table 2 gives an overview of the phenolics content and antioxidant activity of blackberry wine samples. It is evident that the obtained values were not homogeneous.

### 3.1. Phenolics in Blackberry Wine

Phenolics are a group of several thousands of compounds that have an aromatic ring with different numbers and types of functional groups in their structure. It can be seen from the data in Table 2 that the TPI of the analyzed blackberry wines ranged from 33.1 (CBW 1) to 89.5 (OBW 15). This test represents a rapid and straightforward method for the determination of polyphenolics in different wine samples. However, the main disadvantage of this method is the interference of nonphenolic aromatic compounds also present in sample, such as aromatic amino acids, nucleotides, peptides, etc. [35].

TPH (Table 2) in analyzed blackberry wine samples was in a broad range, from 868 mg/L (CBW 1) to 2581 mg/L (OBW 11), with the mean value of all samples being 1966 mg/L. A comparison of the obtained results based on the blackberry cultivation mode did not reveal significant differences of TPH between the two groups of samples, even though the mean value of organic group samples was slightly higher than that of the conventional group (mean_ORG_: 1965 mg/L mean_CON_: 1772 mg/L).

The present results are also comparable to the results of other authors reporting the TPH content of blackberry wines. Čakar et al. [9] used different types of fruit (raspberry, blackberry, chokeberry and blueberry, apple, and cherries) for the production of fruit wines. The TPH contents of two produced blackberry wines were 2230.46 ± 1.55 mg/L, for the blackberry wine without added sugar, and 2326.81 ± 1.27 mg/L for the one with added sugar. Mudnic et al. [4] reported the TPH content of four different blackberry wine samples (1697 ± 20 to 2628 ± 29 mg_GAE_/L), and Johnson and Mejia [8] did so for six blackberry wines (mean TPH value 2212.5 mg/L expressed as ellagic acid equivalents with a wide range), while Kalkan Yildirim [36] reported blackberry wines TPH concentrations of 1232 mg_GAE_/L.

It was well known that determinations of the TPH and TPI suffer from overestimation, due to interferences [35]. Nevertheless, the results obtained by both analyses (TPI and TPH) showed statistically significantly correlation (Table 3).

Tannins are astringent and bitter plant polyphenes that can form complexes with proteins, starch, cellulose and minerals. The concentrations of nontannins in both groups of tested samples oscillated from 666 mg/L (CBW 1) to 2117 mg/L (OBW 15). The obtained results presented in Table 3 indicate a high correlation between the nontannin components and TPI (*r* = 0.8851; *p* < 0.01), as well as TPH (*r* = 0.8961, *p* < 0.01).

Tannins (mainly in the form of condensed tannins) are often present in wine in substantial amounts, especially in red grape wines [28]. The concentration of tannins in analyzed samples ranged from 197 mg/L (OBW 14) to 1158 mg/L (OBW 11), with the mean value of all samples being 562 mg/L. Wine tannins originate from different sources and their concentration is dependent on their content in the fruits used in winemaking (grapes or other) and the extraction process, and on the particular winemaking process and barrels used to age wine [28].

The most common nonflavonoid compounds in wine are derivatives of hydroxycinnamic and hydroxybenzoic acids, which mainly originate from fruit. The lowest and the highest determined concentrations of nonflavonoids were 681 mg/L and 2037 mg/L in samples CBW 1 and OBW 9, respectively. This is consistent with the study of Mudnic et al. [4], that reported concentrations of nonflavonoids in four types of blackberry wines ranging from 773 to 1486 mg/L, expressed as gallic acid equivalents. A statistically significant correlation between the concentrations of nonflavonoids, TPI, TPH and nontannins was established (Table 3).

The concentration of total flavonoids in the analyzed samples of blackberry wine ranged from 161 to 774 mg/L (Table 2). The concentrations of total flavonoids in blackberry wine samples reported in the present study were lower than those reported by Mudnic et al. [4], that were in the range from 924 to 1417 mg/L. Different factors such as temperature, sulfite and ethanol concentration, the type of fermentation vessel, pH, yeast strain, as well as insecticides and herbicides applied during fruit cultivation, may have an impact on the flavonoid content of wine [28]. The mean concentration between the organic and conventional group of samples did not differ significantly (mean_ORG_ = 491 mg/L, mean_CON_: = 405 mg/L), while the difference between their medians (median_ORG_ = 497 mg/L, median_CON_ = 287 mg/L) was significantly different. Furthermore, a correlation analysis showed a statistically significant positive association between the concentration of tannins and flavonoids in all analyzed samples (Table 3).

Anthocyanins are the predominant wine pigments, transferred to wine from both fruit skin and pulp during the maceration process [37]. The concentration oscillated in an extremely wide range, from 5.07 mg/L (CBW 2) to 217 mg/L (CBW 7). This is in accordance with the study of Johnson and Mejia [8], that reported concentrations of total anthocyanins in blackberry wines in the range from 10.71 mg/L to 191.95 mg/L (expressed as cyaniding-3-glucoside equivalents) and an average concentration of 75.56 mg/L. Mudnic et al. [38] also reported an extremely wide range of total anthocyanin values for blackberry wines, i.e., from 13.4 ± 3 to 164 ± 3 mg/L (expressed as malvidin 3-glucoside equivalents), and found it to be comparable to total anthocyanins in red grape wine. When a conventional and an organic group of samples were compared, it could be seen that the difference between the mean (76.2 mg/L) and the median (24.42 mg/L) value was high for the conventional group, while for the organic group, this difference was considerably lower (mean_ORG_ = 53.5 mg/L; median_ORG_ = 49.7 mg/L). In our previous research of blackberry wines originating from three Croatian subregions [3], we reported a statistically significant difference of total anthocyanin concentrations between the samples from three subregions. In contrast, the results of the present study did not indicate a statistically significant difference in total anthocyanins between the two investigated groups of wines, i.e., conventional and organic (Table 2).

Fruit and fruit wines are a rich source of these compounds, such as phenolic acids. In this study, we determined the concentrations of five phenolic acids (gallic, caffeic, chlorogenic, cinnamic, and *p*-coumaric acid) present in blackberry wines using the appropriate HPLC method.

Gallic acid is the dominant polyphenolic compound of blackberry wines; its concentration in the analyzed samples varied over a wide range, i.e., from 23.7 mg/L (CBW 5) to 118 mg/L (OBW 15). The mean value of all the analyzed wines was 69.42 mg/L. By observing the mean values listed in Table 2, it can be seen that gallic acid was present in the organic group of samples in higher amounts (mean_ORG_: 77.9 mg/L) than in the conventional group (mean_CON_: 59.7 mg/L). However, this difference was not statistically significant (*p* ˃ 0.05). Čakar et al. [9] investigated the concentration of gallic acid in blackberry wine samples, and found it to be 92.15 ± 1.29 mg/L and 100.17 ± 1.20 mg/L in samples without and with added sugar, respectively. However, ellagic acid, and not gallic acid, was determined to be the predominant phenolic acid in the analyzed blackberry wines. The contents of ellagic acid were 132.97 ± 1.06 mg/L and 140.20 ± 1.60 mg/L for wine without and with added sugar, respectively.

The concentration of chlorogenic acid, the ester formed between caffeic acid and the 3-hydroxyl of L-quinic acid, in the investigated fruit wines ranged from 1.23 mg/L (OBW 12) to 8.32 mg/L (OBW 14); the average values of this acid did not differ significantly (Table 2).

In contrast, obtained results showed that there was a statistically significant difference (Table 2) in the caffeic acid content between the two groups of samples (mean_ORG_: 3.73 mg/L; mean_CON_: 2.21 mg/L). Caffeic acid concentrations in the analyzed samples ranged from 1.25 mg/L (CBW 7) to 5.05 mg/L (OBW 15); the mean value of all samples was 3.02 mg/L. Čakar et al. [9] reported the concentration of caffeic acid for two analyzed samples of blackberry wine to be 2.44 ± 0.11 mg/L (sample without added sugar) and 3.78 ± 0.08 mg/L (sample with added sugar). The concentration of caffeic acid was correlated with other analyzed bioactve polyphenolic compounds of blackberry wine, and a statistically significant positive correlation was found between caffeic acid and nontannins, gallic acid and *p*-coumaric acid (Table 3), probably as a result of the synthesis of polyphenols in blackberry.

The concentration of *p*-coumaric acid in the investigated fruit wines oscillated from 0.331 mg/L (CBW 5) to 4.06 mg/L (OBW 15). The available literature data show the impact of organic cultivation on the amount of *p*-coumaric acid in fruits [39,40]. The concentration of *p*-coumaric acid (median_CON_: 0.814 mg/L) of the conventional group was statistically significantly lower than that of the organic group (median_ORG_: 2.40 mg/L) (Table 2).

Furthermore, the concentration of cinnamic acid in the aglycon form in all analyzed wine samples was below the LOQ (0.101 mg/L) of the proposed method. The available literature data describe the presence of cinnamic acid in wines as glycosides or in combination with polyphenolic constituents (e.g., anthocyanidins) or some other substances [41,42,43].

Red wine is one of the primary natural sources of *trans*-resveratrol, the most active form of resveratrol, a compound exhibiting anticarcinogenic, anti-inflammatory and antidiabetic properties [11,12,13]. As can be seen from Table 2, the highest concentration of this compound was determined in wine sample OBW 9 (4.00 mg/L), while the lowest was determined in sample CBW 1 (0.415 mg/L). The average value of *trans*-resveratrol was 1.53 mg/L, which is comparable to the results reported by Mudnic et al. [4] for four different blackberry wines; the concentration of *cis*- and *trans*-resveratrol was below 1.5 mg/L. The average value of *trans*-resveratrol in the present study was higher than those reported for Greek red and white wines [44], but slightly lower than that for Spanish red wines [45]. A comparison between the organic and conventional group of samples did not reveal a statistically significant difference in the average concentration of trans-resveratrol in the investigated groups (mean_ORG_ = 1.73 mg/L; mean_CON_ = 1.30 mg/L). On the other hand, Tinttunen and Lehtonen [46] established a positive connection between the fruit (grape) cultivation mode and the concentration of *trans*-resveratrol. They analyzed 58 samples of conventional and organic white and red wines, and concluded that the concentration of *trans*-resveratrol was higher in organic red wines (mean_ORG_: 5.6 mg/L; mean_CON_: 2.9 mg/L) than in conventional red and white wines.

### 3.2. Antioxidant Activity of Blackberry Wines

Since blackberry wine contains a wide variety of free radical-scavenging molecules, such as polyphenols, the aim of this work was also to investigate the antioxidative activity of Croatian blackberry wines. The IC_50%_ values for DPPH and ABTS methods were calculated using the regression equation from the obtained curves; the results are shown in Table 2. The obtained results show that the IC_50%_ of the values obtained by the ABTS method did not differ significantly from the values obtained by the DPPH method. Organic group samples OBW 13, OBW 14 and OBW 15 showed a high antiradical effect, i.e., a low IC_50%_ for the ABTS test. However, the mean value of the organic samples group was 4.05 mg/L, and it was not significantly different from the conventional samples group (mean_CON_ = 4.95 mg/L).

**Figure 2:**
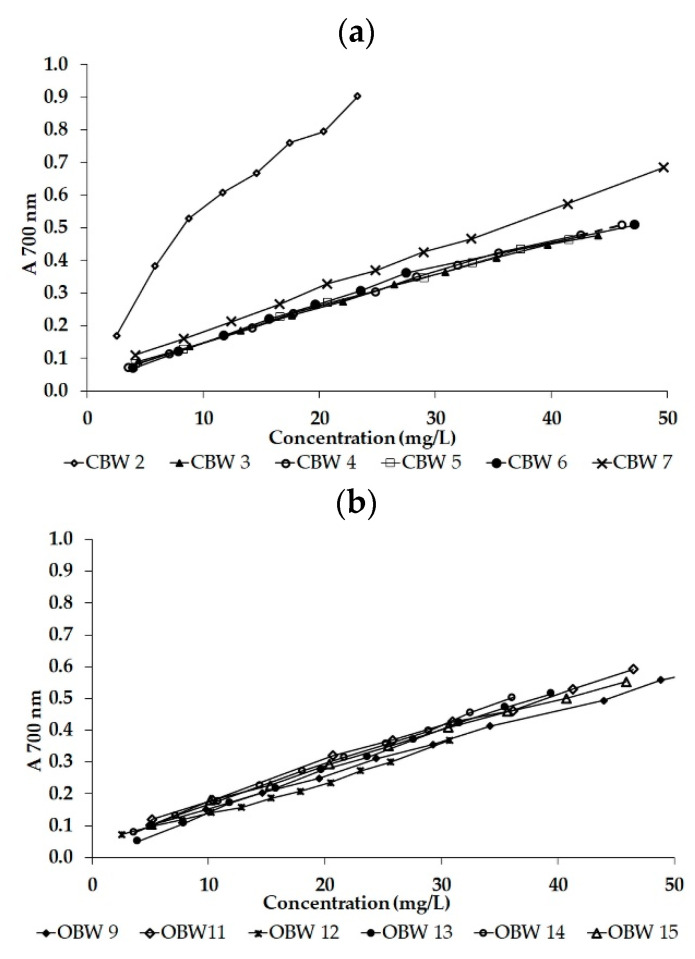
The reductive capacity of the tested conventional (CBW) (**a**) and organic (OBW) (**b**) group BW samples estimated by the RPA method (A 700nm—absorbance at 700 nm).

As an attempt to further elucidate the influence of fruit cultivation mode on the antioxidant activity of blackberry wines, the RPA method was used. Figure 2 shows the reductive ability of the analyzed samples as a function of the concentration of total polyphenols determined by this spectrophotometric method. The increased absorbance of the reaction mixture indicated a higher reductive capacity of the sample, which was estimated at a concentration of 40 mg/L for comparison. The lowest absorbance of the resulting complex was measured in sample OBW 9 (0.44), while the highest was in sample CBW 2 (1.36).

The reductive ability of blackberry wines was evaluated by a molybdenum test based on the reduction of molybdenum (VI) in the presence of antioxidants and the formation of a green molybdenum (V) complex. The results obtained by this method were expressed as vitamin C equivalents (Table 2), and ranged from 2.99 mg/mL (OBW 12) to 9.87 mg/mL (CBW 3). Currently, there is no literature available on the reductive ability of blackberry wines by the molybdenum test; most studies provide data on the evaluation of red wines, grape extracts or other plants [47,48]. Besides, due to the different preparation of the extract and different expression of the results, it is difficult to compare the results of this study with the available literature data.

Furthermore, a comparison of the antioxidant activity results with other compounds present in blackberry wine revealed a statistically significant correlation between the IC_50%_ concentration obtained by the DPPH test, TPI and nonflavonoid fraction of polyphenols (Table 3). A statistical analysis of the obtained results also revealed the existence of a correlation between the concentration of IC_50%_ obtained by the ABTS test and the amount of nontannin polyphenols in blackberry wine. The highest degree of correlation between the reductive capacity estimated by the molybdenum test was observed with the tannins content. The reported results are in agreement with the available literature data for different grape wines and fruit wines [9,49,50].

Finally, the results of this study related to the antioxidant activity are completely in line with our previously published work [3].

### 3.3. Multivariate Analysis

PCA for the model of polyphenol content and antioxidant activity revealed that the first six PCs (out of 14) explain 88.8% of the total variance. These six PCs were used for further analysis.

**Figure 3:**
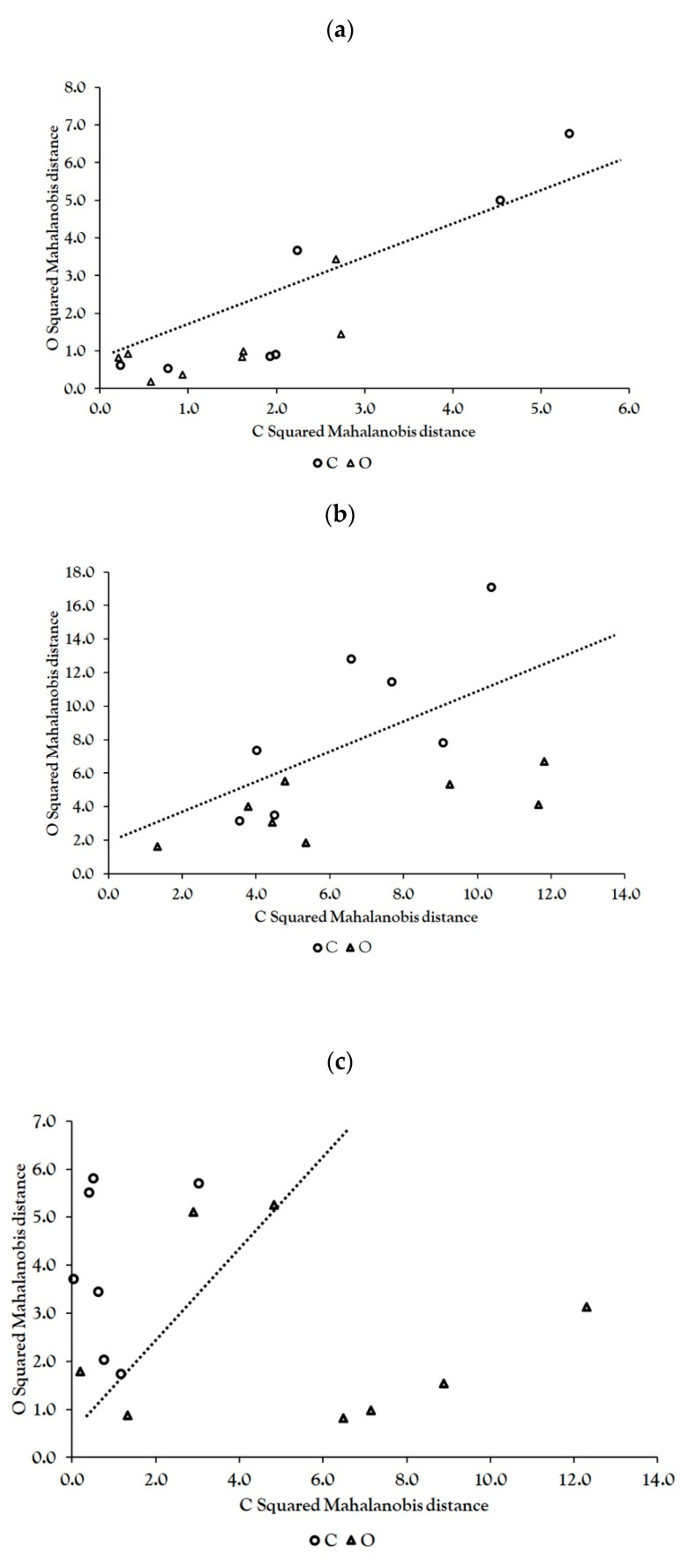
GDA models of polyphenolic content and antioxidant activity for the investigated conventional (CBW) and organic (OBW) group BW samples presented as squared Mahalanobis distances for each group; dotted lines separate predicted groups, and the triangles (OBW) and circles (CBW) represent the original membership to a group; (**a**) GDA1 (**b**) GDA2 and (**c**) GDA3.

As one of the objectives of this study was to try to establish a connection between the polyphenolic content and antioxidant activity and the applied blackberry cultivation method, GDA was used to determine the discriminant power of both the six PCs determined previously and the original dataset (using a backward stepwise procedure to reduce the number of variables). The nonerror rate (NER%) of the GDA1 model of polyphenolic content and antioxidant activity for the original dataset was 60% with the canonical *R* for the model of 0.237 (*p* = 0.707) (Figure 3a). This model, based on the original dataset of polyphenolic content and antioxidant activity, included two variables from the original dataset (ACY and MoT) but did not provide a satisfactory separation of the two applied cultivation methods. The GDA2 model, based on PCs using a backward stepwise procedure, did not include any of the variables, so GDA2 was conducted additionally using all effects analysis, but the NER% was still low (66.6%), with a canonical R for the model of 0.648 and a nonsignificant separation between groups based on the applied cultivation method (*p* = 0.504) (Figure 3b). The GDA3 model that included only two variables from the original dataset that were significantly different between two applied cultivation methods (caffeic acid and *p*-coumaric acid) using all effects analysis still had a moderate NER%, i.e., 80.0%, with canonical R for the model of 0.685 with a significant separation between groups (squared Mahalanobis distances 3.073, *p* = 0.022) (Figure 3c).

## 4. Conclusions

The results of our study show a series of statistically highly significant relationships between the analyzed constituents (caffeic acid and *p*-coumaric acid), which are the result of their synthesis in blackberry fruit and probably the general production process characteristics for all types of wine.

The antioxidant activity of the investigated blackberry wines was proportional to the measured concentrations of both total polyphenolics and individual groups (tannins, nonflavonoids) of these bioactive compounds.

A comparison between the two groups of wine samples—conventional and organic (based on the blackberry cultivation method)—revealed a statistically significant difference in the concentration of caffeic acid and *p*-coumaric acid, with both being higher in samples denoted as organic, i.e., produced from organically grown blackberries.

The observed very wide concentration range of bioactive compounds, such as total polyphenolics, tannins, flavonoids, anthocyanins and gallic acid, could be the result of different starting raw material (i.e., blackberries) quality, but also of the different vinification procedures applied by producers. The latter calls for standardization of the technological process of blackberry wine production in order to balance the composition and improve the quality of the final product.

## Figures and Tables

**Table 1:** HPLC method validation parameters for the determination of six individual phenolic compounds in blackberry wine.

Parameter	Gallic Acid	Chlorogenic Acid	Caffeic Acid	*p*-Coumaric Acid	Cinnamic Acid	*trans*-Resveratrol
t_R_ [min]	9.67	37.81	39.45	52.61	99.20	89.33
λ_max_ [nm]	272.3	328.2	325.4	311.1	280.3	307.2
λ_quant_ [nm]	280	323	323	313	280	280
Calibration interval [mg/L]	1–40	0.25–10	0.50–20	0.50–20	0.50–20	0.50–20
Calibration line (*n* = 6)	y = 1.0764x − 0.2043	y = 0.9439x − 0.1605	y = 1.9619x − 0.1292	y = 2.9848x − 0.0560	y = 3.2168x − 0.0040	y = 1.7736x − 0.2210
Correlation coefficient (*n* = 5)	0.9998	0.9990	0.9990	0.9991	0.9994	0.9996
LOD [mg/L]	0.071	0.064	0.096	0.038	0.046	0.135
LOQ [mg/L]	0.235	0.213	0.165	0.084	0.101	0.450
Intra-day precision standard solution RSD [%] (*n* = 10)	0.64	1.27	1.38	1.51	0.92	1.12
Intra-day precision BW sample RSD [%] (*n* = 10)	0.69	1.47	1.60	1.60	-	1.89

t_R_—retention time; λ_max_—wavelength of maximum absorbance of the compound; λ_quant_—wavelength for the quantitative analyses of the compound; LOD—limit of detection; LOQ—limit of quantitation; RSD—relative standard deviation; BW—blackberry wine.

**Table 2:** Descriptive statistical analysis of polyphenol content and antioxidant activity in conventional and organic BW samples.

Parameter	All Samples		Conventional Group				Organic Group				ANOVA	
	Range	RSD (%)	Mean ± SD	Median	Range of Quantified Values	Interquartile	Mean ± SD	Median	Range of Quantified Values	Interquartile	*F*	*p*
TPI	33.1–89.5	0.01–0.83	55.9 ± 14.0	56.2	33.1–75.2	47.8–68.0	69.3 ± 14.1	73.0	44.4–89.5	59.3–78.0	3.3849	0.0887
TPH (mg/L)	868–2581	0.02–9.26	1772 ± 469	1965	868–2202	1455–2075	2136 ± 466	2206	1280–2581	1838–2541	2.2599	0.1567
Total tannins (mg/L)	197–1158	– ^1^	538 ± 325	491	202–1128	307–804	583 ± 300	534	197–1158	362–756	0.0779	0.7845
Nontannins (mg/L)	666–2117	0.05-5.70	1234 ± 307	1271	666–1568	1074–1474	1404 ± 347	1437	666–2117	1348–1751	3.4938	0.0843
Total flavonoids (mg/L)	161–774	– ^1^	405 ± 247	287	161–774	187–699	491 ± 114	497	307–685	412–563	0.7745	0.3948
Nonflavonoids (mg/L)	681–2037	0.05–5.27	1367 ± 387	1452	681–1804	1168–1804	1645 ± 415	1696	842–2037	1450–2008	1.7871	0.2042
ACY (mg/L)	5.07–217	0.41–5.46	76.2 ± 87.4	24.2	5.34–217	7.11–168	53.5 ± 29.5	49.7	5.07–97.9	33.6–79.6	0.4825	0.4995
Gallic acid (mg/L)	23.7–118	0.10–4.84	59.7 ± 40.7	31.4	23.7–116	26.5–101.2	77.9 ± 26.5	72.4	49.9–118	52.2–104	1.0816	0.3173
Chlorogenic acid (mg/L)	1.23–8.32	0.34–2.93	3.46 ± 1.31	3.14	1.67–5.15	2.23–4.96	3.59 ± 2.09	3.21	1.23-8.32	2.47–3.93	0.0209	0.8872
Caffeic acid (mg/L)	1.25–5.05	0.07–3.63	2.21 ± 0.84	2.05	1.25–3.33	1.47–3.08	3.73 ± 1.22	4.09	1.40–5.05	2.84–4.75	7.6597	0.0159
*p*-Coumaric acid (mg/L)	0.331–4.06	0.22–4.41	0.814 ± 0.426	0.660	0.331–1.21	0.361–1.21	2.40 ± 1.23	2.39	0.881–4.06	1.25–3.41	10.3478	0.0067
*trans*-Resveratrol (mg/L)	0.415–4.00	0.24–4.93	1.30 ± 0.75	1.32	0.415–2.55	0.666–1.82	1.73 ± 1.10	1.51	0.722–4.00	0.859–2.28	0.7566	0.4002
Cinnamic acid	<LOD (0.046 mg/L)											
*Antioxidant activity*												
DPPH (mg/L)	4.39–6.69	0.19–8.67	5.43 ± 0.65	5.62	4.53–6.27	4.57–5.84	5.20 ± 0.80	4.91	4.39–6.69	4.67–5.92	0.3713	0.5528
ABTS (mg/L)	1.23–7.77	0.45–7.98	4.95 ± 1.60	5.38	2.81–7.00	2.82–5.95	4.05 ± 2.15	4.20	1.23–7.77	2.19–5.46	0.8224	0.3809
RPA ^2^	0.44–1.36	0.02–5.69	0.61 ± 0.33	0.46	0.45–1.36	0.44–0.56	0.51 ± 0.45	0.51	0.44–0.57	0.46–0.54	0.7961	0.3885
MoT (mg/L)	2.99–9.87	0.03–7.44	5.95 ± 2.13	5.60	3.79–9.87	3.80–7.10	5.25 ± 2.37	4.71	2.99–9.39	3.10–7.09	0.3637	0.5569

Cinnamic acid was below the LOD value. No significant differences were found between the triplicates for all determinations. LOD—limit of detection; TPI—total polyphenol index; TPH—total polyphenolic compounds; ACY—total monomeric anthocyanins; DPPH—IC_50%_ values for DPPH method; ABTS—IC_50%_ values for ABTS method; RPA—Reducing power assay; MoT—Molybdenum test; ^1^ The values were calculated from Equation: Total tannins = TPH—Nontannins; Total flavonoids = TPH—Nonflavonoid. ^2^ RPA of the samples was evaluated at a concentration of total polyphenols (40 mg/L).

**Table 3:** Statistically significant correlations (*p* < 0.01) between the analyzed parameters and compounds of blackberry wines.

Parameter		*r*	*p*
Non–flavonoids	TPI	0.9486	*p* = 0.000
Non–flavonoids	TPH	0.9281	*p* = 0.000
TPH	TPI	0.8961	*p* = 0.000
Non–tannins	TPI	0.8851	*p* = 0.000
Non–flavonoids	Nontannins	0.8707	*p* = 0.000
Non–tannins	TPH	0.7890	*p* = 0.000
*p*-Coumaric acid	TPI	0.7702	*p* = 0.001
*p*-Coumaric acid	Non–tannins	0.7655	*p* = 0.001
*p*-Coumaric acid	Caffeic acid	0.7580	*p* = 0.001
Caffeic acid	Non–tannins	0.7567	*p* = 0.001
Total flavonoids	Total tannins	0.7493	*p* = 0.001
DPPH	TPI	−0.7443	*p* = 0.001
MoT	Total tannins	0.7423	*p* = 0.002
DPPH	Non–flavonoids	−0.7110	*p* = 0.003
ABTS	Non–tannins	−0.7077	*p* = 0.003
Caffeic acid	Gallic acid	0.7060	*p* = 0.003

TPI—total polyphenol index; TPH—total polyphenolic compounds; DPPH—DPPH method; ABTS—ABTS method; MoT—Molybdenum test
